# Crystal structure of 5,5-bis­(4-methyl­benz­yl)pyrimidine-2,4,6(1*H*,3*H*,5*H*)-trione monohydrate

**DOI:** 10.1107/S205698901402619X

**Published:** 2015-01-01

**Authors:** Bhaskarachar Ravi Kiran, Parameshwar Adimule Suchetan, Hosamani Amar, Giriyapura R Vijayakumar

**Affiliations:** a Prof. CNR Rao Centre for Advanced Materials and Department of Chemistry, UCS, Tumkur University, Tumkur 572 103, India; b Solid State and Structural Chemistry Unit, Indian Institute of Science, Bangalore 560 012, India

**Keywords:** crystal structure, hydrogen-bonded sheets, pyrimidine, C—H⋯π inter­actions, Knoevenagel condensation, three-dimensional structure

## Abstract

The asymmetric unit of the title compound, C_20_H_20_N_2_O_3_·H_2_O, contains two independent mol­ecules (*A* and *B*), with similar conformations and two independent water mol­ecules. In the crystal, N—H⋯O and O_water_—H⋯O hydrogen bonds link all moieties into two crystallographically independent kinds of sheets parallel to the *ac* plane. These independent sheets, each containing either *A* or *B* mol­ecules, are further alternately stacked along the *b* axis and inter­connected *via* C—H⋯π_ar­yl_ inter­actions.

## Abstract 2

Reaction of 4-methyl benzyl chloride with barbituric acid gave 5,5-bis­(4-methyl­benz­yl)pyrimidine-2,4,6(1*H*,3*H*,5*H*)-trione, which crystallized with two mol­ecules in its asymmetric unit along with two solvent water mol­ecules. A hydrogen-bonded sheet is formed by a combination of N—H⋯O and O_water_—H⋯O hydrogen bonds, which are further inter­connected by C—H⋯π_ar­yl_ inter­actions, leading to a three-dimensional supra­molecular architecture.

## Chemical Context   

Barbituric acid and its derivatives have historically been classified as compounds which act on the central nervous system (Barbachyn *et al.*, 2007 ▸). These compounds have been widely used as therapeutic drugs such as anxiolytics, sedatives, hypnotics and anti-convulsants (Coupey, 1997 ▸). Recent investigations on barbituric acid derivatives revealed the applications of these compounds as anti­bacterial (Yilmaz *et al.*, 2006 ▸; Sweidan *et al.*, 2011 ▸), anti-viral (Clercq, 1986*a* ▸,*b* ▸; Baba *et al.*, 1987 ▸), analgesic (Vida *et al.*, 1975 ▸), anti-hypertensive (Bassin & Bleck, 2008 ▸) and as anti-cancer (Humar *et al.*, 2004 ▸; Singh *et al.*, 2009 ▸) agents. 5-Fluoro­uracil is a barbituric acid analogue, which has been widely employed as a clinically useful anti-cancer drug (Heidelberger & Arafield, 1963 ▸).
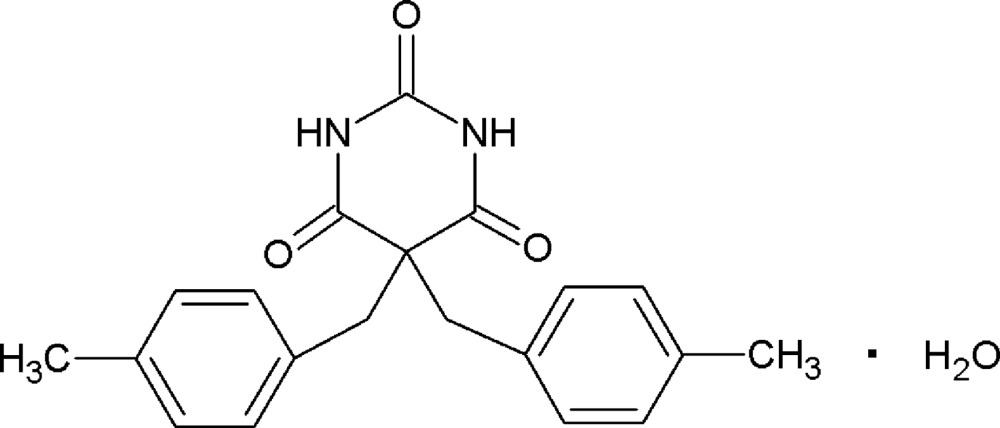


Inspired by the above facts, the title compound was synthesized by Knoevenagel condensation reaction (Prajapati and Gohain, 2006 ▸). A double-benzyl­ated product of barbituric acid was obtained by using two equivalents of 4-methyl benzyl chloride in the presence of catalytic amounts of 1,8-di­aza­bicyclo­undec-7-ene (DBU) and solvent aceto­nitrile. The obtained compound was characterized by ^1^H-NMR and mass spectroscopy. We report herein on its crystal structure.

## Structural commentary   

**Figure 1:**
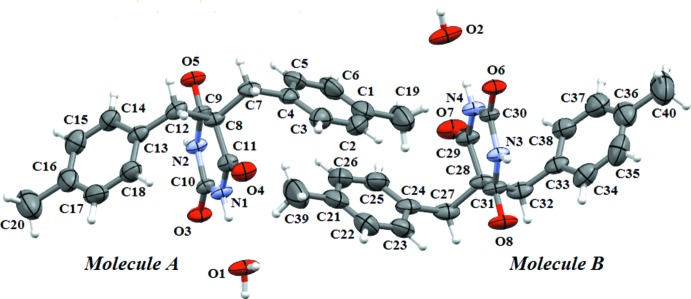
A view of (I), showing the atom labelling. Displacement ellipsoids are drawn at the 50% probability level.

The title compound (I) (Fig. 1 ▸) crystallizes with two mol­ecules, *A* and *B*, in the asymmetric unit along with two water mol­ecules of crystallization. In both mol­ecules, the pyrimidine rings are nearly planar [r.m.s. deviations of 0.039 and 0.040 Å] and can be considered as a pseudo-mirror plane for each mol­ecule. In *A*, the benzene rings form dihedral angles of 49.70 (17) and 51.66 (17)° with the pyrimidine ring and are inclined each to other by 62.9 (2)°. In *B*, the corresponding angles are 50.44 (18), 69.90 (19) and 59.8 (2)°, respectively. In the related compound 5,5-di­benzyl­barbituric acid monohydrate (II) (Bhatt *et al.*, 2007 ▸), which crystallizes with one independent mol­ecule and one water molecule in the asymmetric unit, the dihedral angles between the pyrimidine and two benzene rings are 54.09 (11) and 62.71 (11)°.

## Supra­molecular features   

**Figure 2:**
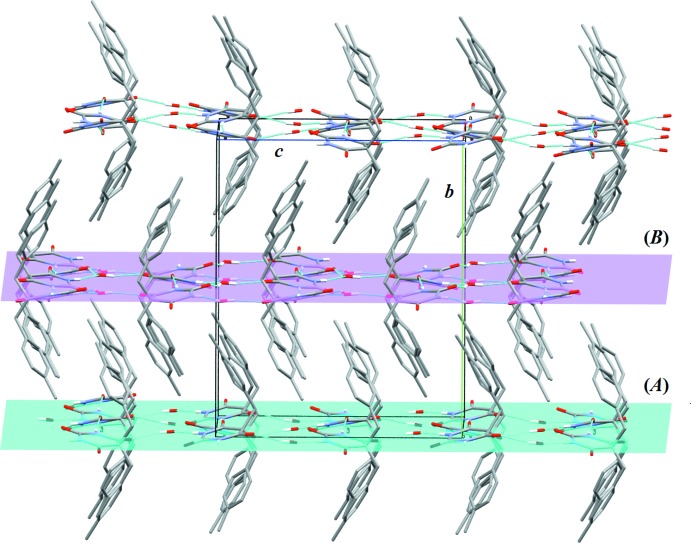
A portion of the crystal packing viewed along [100] and showing two kinds of hydrogen-bonded (thin blue lines) sheets, each containing either *A* or *B* mol­ecules. H atoms not involved in hydrogen bonding have been omitted for clarity.

**Figure 3:**
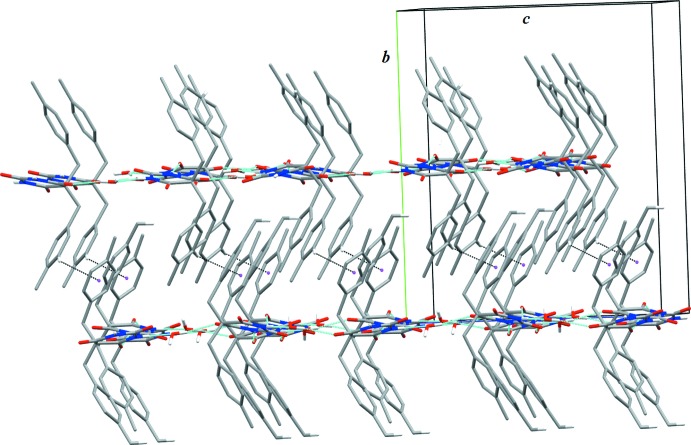
A portion of the crystal packing viewed along [100] and showing C—H⋯π inter­actions as dotted lines. Magenta dots show the centroids of aryl rings. Thin blue lines denote hydrogen bonds. H atoms not involved in inter­molecular inter­actions were omitted for clarity.

In the first level of packing, the independent mol­ecules are linked directly to their symmetry equivalents *via* strong N2—H*N*2⋯O4 (*A*) and N3—H*N*3⋯O7 (*B*) hydrogen bonds (Table 1 ▸), forming chains running along *a-*axis direction. Thus, the graph set motif is *C*(6)*C*(6). Six chains pass through the unit cell. These chains are linked *via* water mol­ecules through N1—H1⋯O1 and O1—H1*B*⋯O5 (for *A*) and N4—H*N*4⋯O2 and O2—H2*A*⋯O8 (for *B*) hydrogen bonds (Table 1 ▸), each forming graph set motif *D*(2). In addition, the symmetry-dependent parallel chains are inter­connected *via* bridging water mol­ecules through O—H⋯O3 and O1—HI*B*⋯O5 (for *A*) and O2—H2*B*⋯O6 and O2—H2*A*⋯O8 (for *B*) hydrogen bonds (Table 1 ▸), forming sheets parallel to the *ac* plane (Fig. 2 ▸). The alternate sheets formed by *A* or *B* mol­ecules and water mol­ecules are inter­connected *via* C—H⋯π inter­actions (Fig. 3 ▸, Table 1 ▸), thus forming a three-dimensional structure.

There are several inter­esting differences between the two chemically closely related structures (I) and (II) (differing only by a methyl group on the two benzene rings). Firstly, (I) crystallizes in the ortho­rhom­bic space group *Pca*2_1_, whereas (II) crystallizes in the monoclinic space group *P*2_1_/*n*. Secondly, (I) crystallizes with two mol­ecules in its asymmetric unit, while (II) crystallizes with one independent mol­ecule. Lastly, in the crystal of compound (II), hydrogen bonding leads to a two-dimensional network in contrast to the three-dimensional architecture formed in (I).

## Synthesis and crystallization   

To an ice-cooled stirring solution of acetonitrile (5 ml), 4-methyl benzyl chloride (0.5 g, 0.0035 mol), 1,8-diazabicycloundec-7-ene (DBU) (0.5 g, 0.0035 mol) and barbituric acid (0.22 g, 0.0017 mol) were added. The reaction mixture was stirred to the room temperature and then refluxed for 8 h. Thin-layer chromatography showed the absence of any starting material. The reaction mixture was cooled and poured into ice-cold water. The solid obtained was extracted with ethyl acetate and the organic layer was washed with saturated ammonium chloride solution and dried over anhydrous sodium sulphate. The solvent was removed under reduced pressure to give the title compound as a white solid (Yield 0.54 g, 91.83%).

Colourless prisms of the title compound suitable for diffraction studies were grown from an ethyl acetate–petroleum ether solvent system in the ratio 2.5:7.5, by the solvent evaporation technique.

## Refinement   

Crystal data, data collection and structure refinement details are summarized in Table 2 ▸. The water H atoms were located in a difference Fourier map and freely refined. The amino and C-bound H atoms were fixed geometrically (N—H = 0.86, C—H = 0.93–0.97 Å) and allowed to ride on their parent atoms with 1.5*U*_eq_(C) for methyl H atoms and = 1.2*U*_eq_(N,C) for other H atoms.

## Supplementary Material

Crystal structure: contains datablock(s) I, global. DOI: 10.1107/S205698901402619X/cv5478sup1.cif

Structure factors: contains datablock(s) I. DOI: 10.1107/S205698901402619X/cv5478Isup2.hkl

Supporting information file. DOI: 10.1107/S205698901402619X/cv5478Isup3.cml

CCDC reference: 1036677

Additional supporting information:  crystallographic information; 3D view; checkCIF report

## Figures and Tables

**Table 1:** Hydrogen-bond geometry (, ) *Cg* is the centroid of the C13C18 benzene ring.

*D*H*A*	*D*H	H*A*	*D* *A*	*D*H*A*
N1H*N*1O1	0.86	1.94	2.787(4)	167
O1H1*A*O3^i^	0.84(3)	2.13(3)	2.949(3)	162
O1H1*B*O5^ii^	0.90(3)	1.90(3)	2.794(4)	175
N2H*N*2O4^iii^	0.86	1.94	2.767(2)	162
O2H2*A*O8^iv^	0.86(6)	1.88(6)	2.739(4)	177
O2H2*B*O6^v^	0.82(3)	2.16(3)	2.961(3)	169
N3H*N*3O7^vi^	0.86	1.90	2.739(2)	164
N4H*N*4O2	0.86	1.92	2.761(4)	166
C22H22*Cg* ^vii^	0.93	2.97	3.5693	124

Symmetry codes: (i) 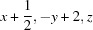; (ii) 
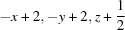
; (iii) 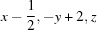; (iv) 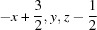; (v) 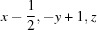; (vi) 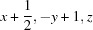; (vii) 
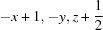
.

**Table 2:** Experimental details

Crystal data	
Chemical formula	C_20_H_20_N_2_O_3_H_2_O
*M*_r_	354.40
Crystal system, space group	Orthorhombic, *P* *c* *a*2_1_
Temperature (K)	296
*a*, *b*, *c* ()	13.0920(17), 19.198(3), 15.827(2)
*V* (^3^)	3978.1(9)
*Z*	8
Radiation type	Mo *K*
(mm^1^)	0.08
Crystal size (mm)	0.39 0.27 0.19

Data collection	
Diffractometer	Bruker APEXII
Absorption correction	Multi-scan (*SADABS*; Bruker, 2009 ▸)
*T*_min_, *T*_max_	0.973, 0.984
No. of measured, independent and observed [*I* > 2(*I*)] reflections	61392, 8543, 4909
*R*_int_	0.057
(sin /)_max_ (^1^)	0.649

Refinement	
*R*[*F* ^2^ > 2(*F* ^2^)], *wR*(*F* ^2^), *S*	0.049, 0.135, 0.98
No. of reflections	8543
No. of parameters	489
No. of restraints	29
H-atom treatment	H atoms treated by a mixture of independent and constrained refinement
_max_, _min_ (e ^3^)	0.20, 0.14
Absolute structure	Flack (1983 ▸), 4587 Friedel pairs
Absolute structure parameter	0.1(1)

Computer programs: *APEX2*, *SAINT-Plus* and *XPREP* (Bruker, 2009 ▸), *SHELXS97* and *SHELXL97* (Sheldrick, 2008 ▸) and *Mercury* (Macrae *et al.*, 2008 ▸).

## supplementary crystallographic information

### Crystal data

**Table:**

C_20_H_20_N_2_O_3_·H_2_O	Prism
*M**_r_* = 354.40	*D*_x_ = 1.183 Mg m^−^^3^
Orthorhombic, *P**c**a*2_1_	Melting point: 443 K
Hall symbol: P 2c -2ac	Mo *K*α radiation, λ = 0.71073 Å
*a* = 13.0920 (17) Å	Cell parameters from 138 reflections
*b* = 19.198 (3) Å	θ = 1.9–27.5°
*c* = 15.827 (2) Å	µ = 0.08 mm^−^^1^
*V* = 3978.1 (9) Å^3^	*T* = 296 K
*Z* = 8	Prism, colourless
*F*(000) = 1504	0.39 × 0.27 × 0.19 mm

### Data collection

**Table:**

Bruker APEXII diffractometer	4909 reflections with *I* > 2σ(*I*)
Radiation source: fine-focus sealed tube	*R*_int_ = 0.057
Graphite monochromator	θ_max_ = 27.5°, θ_min_ = 1.9°
phi and φ scans	*h* = −16→16
Absorption correction: multi-scan (*SADABS*; Bruker, 2009)	*k* = −24→24
*T*_min_ = 0.973, *T*_max_ = 0.984	*l* = −19→20
61392 measured reflections	1 standard reflections every 1 reflections
8543 independent reflections	intensity decay: 1%

### Refinement

**Table:**

Refinement on *F*^2^	Secondary atom site location: difference Fourier map
Least-squares matrix: full	Hydrogen site location: inferred from neighbouring sites
*R*[*F*^2^ > 2σ(*F*^2^)] = 0.049	H atoms treated by a mixture of independent and constrained refinement
*wR*(*F*^2^) = 0.135	*w* = 1/[σ^2^(*F*_o_^2^) + (0.0724*P*)^2^] where *P* = (*F*_o_^2^ + 2*F*_c_^2^)/3
*S* = 0.98	(Δ/σ)_max_ < 0.001
8543 reflections	Δρ_max_ = 0.20 e Å^−^^3^
489 parameters	Δρ_min_ = −0.14 e Å^−^^3^
29 restraints	Absolute structure: Flack (1983), 4587 Friedel pairs
Primary atom site location: structure-invariant direct methods	Absolute structure parameter: 0.1 (1)

### Special details

**Table:**

Experimental. All the solvents employed were of analytical grade. Starting materials and reagents were purchased from Sigma Chemical co. (Saint Louis, USA). The reaction progress was monitored by thin layer chromatography using TLC Silica gel 60 F254 (Merck), and spots were visualized by using ultraviolet light of 254 nm. Melting point was determined by using open capillary and uncorrected value is given. 1*H*-NMR spectrum was recorded on Jeol-400 MHz NMR instrument using CDCl3/DMSO-d6 as solvent. Chemical shift values were expressed in δ (p.p.m.) relative to tetramethylsilane (TMS) as an internal reference standard. Mass spectrum of the compound was recorded on Shimadzu LC-2010EV with ESI probe.1*H*-NMR spectral data of I clearly indicated the formation of I. In the spectrum, a singlet at δ (p.p.m.) value 2.23 corresponds to the two *para*-methyl groups, while, a singlet signal at δ (p.p.m.) value 3.22 is for four alkyl protons (two CH2 groups) and two doublets at 6.93 and 7.07 corresponds to the eight aromatic protons. Two NH protons of the barbituric acid moiety appeared at δ value 7.85 as a singlet. Mass spectrum of I gave a peak with (m/*z*) value = 335.0 which exactly matches with its calculated mass.
Geometry. All s.u.'s (except the s.u. in the dihedral angle between two l.s. planes) are estimated using the full covariance matrix. The cell s.u.'s are taken into account individually in the estimation of s.u.'s in distances, angles and torsion angles; correlations between s.u.'s in cell parameters are only used when they are defined by crystal symmetry. An approximate (isotropic) treatment of cell s.u.'s is used for estimating s.u.'s involving l.s. planes.
Refinement. Refinement of *F*^2^ against ALL reflections. The weighted *R*-factor *wR* and goodness of fit *S* are based on *F*^2^, conventional *R*-factors *R* are based on *F*, with *F* set to zero for negative *F*^2^. The threshold expression of *F*^2^ > 2σ(*F*^2^) is used only for calculating *R*-factors(gt) *etc*. and is not relevant to the choice of reflections for refinement. *R*-factors based on *F*^2^ are statistically about twice as large as those based on *F*, and *R*- factors based on ALL data will be even larger.

### Fractional atomic coordinates and isotropic or equivalent isotropic displacement parameters (Å^2^)

**Table:**

	*x*	*y*	*z*	*U*_iso_*/*U*_eq_	
C6	0.8772 (3)	0.7536 (2)	0.0448 (3)	0.1148 (12)	
H6	0.8098	0.7382	0.0448	0.138*	
C19	0.9200 (4)	0.6505 (2)	0.1358 (3)	0.174 (2)	
H19A	0.9340	0.6568	0.1949	0.262*	
H19B	0.9595	0.6122	0.1147	0.262*	
H19C	0.8487	0.6409	0.1281	0.262*	
C39	0.8413 (4)	0.8470 (2)	0.2588 (3)	0.205 (3)	
H39A	0.8257	0.8861	0.2944	0.308*	
H39B	0.8045	0.8512	0.2066	0.308*	
H39C	0.9133	0.8461	0.2474	0.308*	
C40	0.7798 (5)	0.1883 (2)	0.2643 (4)	0.192 (2)	
H40A	0.8105	0.1576	0.3049	0.288*	
H40B	0.8263	0.1956	0.2182	0.288*	
H40C	0.7179	0.1678	0.2434	0.288*	
H2B	0.520 (2)	0.5106 (14)	0.109 (2)	0.088 (10)*	
H2A	0.583 (3)	0.513 (2)	0.036 (4)	0.156 (19)*	
H1A	1.225 (2)	0.9802 (12)	0.272 (2)	0.092 (10)*	
H1B	1.1706 (19)	1.0021 (12)	0.342 (2)	0.070 (8)*	
C1	0.9482 (4)	0.7161 (2)	0.0883 (3)	0.1193 (12)	
C2	1.0471 (4)	0.7405 (2)	0.0846 (2)	0.1205 (13)	
H2	1.0982	0.7152	0.1115	0.145*	
C3	1.0739 (3)	0.8016 (2)	0.0421 (2)	0.1027 (11)	
H3	1.1413	0.8168	0.0427	0.123*	
C4	1.0001 (2)	0.83994 (14)	−0.00130 (15)	0.0727 (7)	
C5	0.9012 (2)	0.81420 (17)	0.0001 (2)	0.0877 (9)	
H5	0.8500	0.8376	−0.0291	0.105*	
C7	1.02806 (17)	0.90622 (13)	−0.04658 (16)	0.0715 (7)	
H7A	0.9901	0.9082	−0.0992	0.086*	
H7B	1.1001	0.9044	−0.0608	0.086*	
C8	1.00757 (17)	0.97436 (13)	0.00349 (16)	0.0563 (6)	
C9	0.89331 (16)	0.98272 (13)	0.01741 (17)	0.0522 (6)	
C10	0.91876 (18)	1.00450 (11)	0.1679 (2)	0.0510 (6)	
C11	1.06796 (15)	0.97321 (12)	0.08499 (16)	0.0561 (6)	
C12	1.04400 (17)	1.03834 (13)	−0.04920 (18)	0.0678 (7)	
H12A	1.1174	1.0350	−0.0565	0.081*	
H12B	1.0132	1.0356	−0.1048	0.081*	
C13	1.01968 (17)	1.10829 (15)	−0.01215 (17)	0.0685 (7)	
C14	0.9285 (2)	1.14188 (16)	−0.0302 (2)	0.0845 (8)	
H14	0.8828	1.1210	−0.0673	0.101*	
C15	0.9035 (3)	1.2049 (2)	0.0051 (3)	0.1032 (11)	
H15	0.8415	1.2257	−0.0085	0.124*	
C16	0.9688 (4)	1.23778 (19)	0.0602 (3)	0.1149 (12)	
C17	1.0608 (4)	1.2054 (2)	0.0784 (3)	0.1245 (14)	
H17	1.1066	1.2268	0.1152	0.149*	
C18	1.0859 (2)	1.14120 (18)	0.0425 (2)	0.0941 (9)	
H18	1.1481	1.1204	0.0556	0.113*	
C20	0.9419 (4)	1.3093 (2)	0.0984 (4)	0.180 (2)	
H20A	0.9712	1.3131	0.1538	0.270*	
H20B	0.8690	1.3139	0.1022	0.270*	
H20C	0.9687	1.3456	0.0629	0.270*	
C21	0.8100 (4)	0.7794 (2)	0.3035 (3)	0.1294 (14)	
C22	0.8734 (3)	0.7433 (2)	0.3546 (3)	0.1207 (12)	
H22	0.9391	0.7598	0.3642	0.145*	
C23	0.8418 (2)	0.68184 (19)	0.3932 (2)	0.0984 (11)	
H23	0.8869	0.6585	0.4287	0.118*	
C24	0.7465 (2)	0.65484 (16)	0.38039 (18)	0.0820 (8)	
C25	0.6804 (3)	0.6924 (2)	0.3298 (2)	0.1110 (12)	
H25	0.6142	0.6764	0.3210	0.133*	
C26	0.7127 (4)	0.7546 (2)	0.2916 (3)	0.1376 (16)	
H26	0.6674	0.7794	0.2577	0.165*	
C27	0.71252 (17)	0.58865 (16)	0.42374 (18)	0.0824 (9)	
H27A	0.6397	0.5916	0.4342	0.099*	
H27B	0.7464	0.5857	0.4781	0.099*	
C28	0.73450 (17)	0.52040 (14)	0.37356 (17)	0.0655 (7)	
C29	0.67384 (16)	0.52234 (14)	0.29184 (18)	0.0635 (6)	
C30	0.82374 (18)	0.49247 (12)	0.20860 (19)	0.0514 (6)	
C31	0.84770 (17)	0.51243 (15)	0.35966 (18)	0.0630 (7)	
C32	0.69710 (19)	0.45597 (16)	0.42547 (17)	0.0812 (8)	
H32A	0.7295	0.4571	0.4806	0.097*	
H32B	0.6240	0.4601	0.4342	0.097*	
C33	0.7185 (2)	0.38700 (16)	0.38549 (18)	0.0784 (8)	
C34	0.8099 (2)	0.3523 (2)	0.3962 (2)	0.1020 (10)	
H34	0.8596	0.3716	0.4311	0.122*	
C35	0.8296 (4)	0.2887 (3)	0.3557 (4)	0.1307 (16)	
H35	0.8928	0.2673	0.3622	0.157*	
C36	0.7556 (5)	0.2579 (2)	0.3061 (3)	0.1243 (12)	
C37	0.6641 (4)	0.2918 (2)	0.2993 (3)	0.1226 (13)	
H37	0.6127	0.2711	0.2676	0.147*	
C38	0.6443 (2)	0.3541 (2)	0.3365 (2)	0.0993 (10)	
H38	0.5808	0.3750	0.3293	0.119*	
N1	1.01988 (13)	0.98950 (10)	0.15858 (17)	0.0572 (6)	
HN1	1.0568	0.9904	0.2035	0.069*	
N2	0.85962 (14)	0.99882 (10)	0.09608 (16)	0.0534 (5)	
HN2	0.7952	1.0062	0.1017	0.064*	
N3	0.88084 (14)	0.49787 (10)	0.28006 (15)	0.0558 (6)	
HN3	0.9455	0.4913	0.2743	0.067*	
N4	0.72171 (13)	0.50664 (10)	0.21786 (16)	0.0584 (6)	
HN4	0.6847	0.5055	0.1730	0.070*	
O1	1.16630 (18)	0.99390 (14)	0.2863 (2)	0.0976 (8)	
O2	0.57806 (19)	0.51299 (15)	0.0904 (2)	0.1084 (10)	
O3	0.88202 (13)	1.02084 (10)	0.23583 (12)	0.0709 (5)	
O4	1.15820 (10)	0.95863 (10)	0.08464 (13)	0.0775 (5)	
O5	0.83276 (12)	0.97775 (9)	−0.04055 (11)	0.0726 (5)	
O6	0.86065 (13)	0.47823 (10)	0.14088 (12)	0.0709 (5)	
O7	0.58342 (12)	0.53645 (12)	0.29178 (14)	0.0953 (6)	
O8	0.90867 (13)	0.51705 (12)	0.41774 (13)	0.0874 (6)	

### Atomic displacement parameters (Å^2^)

**Table:**

	*U*^11^	*U*^22^	*U*^33^	*U*^12^	*U*^13^	*U*^23^
C6	0.137 (3)	0.088 (3)	0.119 (3)	−0.038 (2)	−0.003 (2)	−0.026 (2)
C19	0.312 (6)	0.093 (3)	0.119 (3)	−0.044 (3)	0.006 (4)	−0.005 (2)
C39	0.362 (8)	0.094 (3)	0.159 (4)	−0.046 (4)	0.014 (5)	−0.006 (3)
C40	0.294 (7)	0.109 (3)	0.172 (5)	0.019 (4)	0.044 (5)	0.025 (3)
C1	0.190 (4)	0.088 (3)	0.080 (2)	−0.001 (3)	−0.010 (3)	−0.019 (2)
C2	0.179 (4)	0.103 (3)	0.079 (2)	0.028 (3)	−0.022 (3)	0.003 (2)
C3	0.113 (2)	0.108 (3)	0.087 (2)	0.022 (2)	−0.009 (2)	−0.009 (2)
C4	0.0893 (18)	0.0841 (18)	0.0448 (15)	0.0125 (16)	0.0001 (14)	−0.0115 (14)
C5	0.094 (2)	0.087 (2)	0.082 (2)	−0.0126 (17)	−0.0143 (17)	−0.0221 (18)
C7	0.0651 (14)	0.105 (2)	0.0445 (16)	0.0115 (13)	0.0061 (12)	−0.0133 (15)
C8	0.0431 (11)	0.0879 (17)	0.0379 (13)	−0.0012 (12)	0.0014 (10)	0.0050 (13)
C9	0.0401 (12)	0.0792 (15)	0.0375 (16)	−0.0035 (10)	−0.0045 (11)	0.0052 (11)
C10	0.0432 (12)	0.0672 (15)	0.0426 (18)	−0.0026 (9)	0.0024 (13)	0.0077 (11)
C11	0.0365 (11)	0.0798 (16)	0.0520 (15)	−0.0043 (11)	0.0038 (11)	0.0030 (13)
C12	0.0529 (12)	0.097 (2)	0.0537 (16)	−0.0022 (12)	0.0092 (12)	0.0155 (15)
C13	0.0573 (13)	0.094 (2)	0.0543 (18)	−0.0113 (13)	−0.0003 (12)	0.0191 (15)
C14	0.0796 (18)	0.086 (2)	0.088 (2)	−0.0055 (15)	−0.0074 (15)	0.0160 (17)
C15	0.101 (2)	0.099 (3)	0.110 (3)	−0.002 (2)	0.006 (2)	0.029 (2)
C16	0.172 (4)	0.086 (3)	0.086 (3)	0.002 (3)	0.017 (3)	0.014 (2)
C17	0.178 (4)	0.114 (3)	0.082 (3)	−0.035 (3)	−0.038 (3)	0.011 (2)
C18	0.095 (2)	0.098 (2)	0.089 (2)	−0.0124 (18)	−0.0202 (17)	0.0148 (18)
C20	0.275 (6)	0.117 (3)	0.148 (4)	0.007 (3)	0.036 (4)	−0.011 (3)
C21	0.192 (4)	0.114 (3)	0.083 (3)	−0.010 (3)	−0.015 (3)	−0.039 (2)
C22	0.140 (3)	0.099 (3)	0.123 (3)	−0.013 (2)	−0.010 (3)	−0.030 (3)
C23	0.095 (2)	0.108 (3)	0.091 (3)	0.004 (2)	−0.0182 (18)	−0.036 (2)
C24	0.0795 (17)	0.115 (2)	0.0511 (16)	0.0048 (17)	−0.0033 (15)	−0.0236 (17)
C25	0.114 (2)	0.130 (3)	0.089 (3)	0.009 (2)	−0.034 (2)	−0.026 (2)
C26	0.203 (5)	0.109 (3)	0.100 (3)	0.015 (3)	−0.054 (3)	−0.018 (3)
C27	0.0600 (15)	0.135 (3)	0.0525 (18)	0.0095 (15)	0.0060 (12)	−0.0218 (18)
C28	0.0446 (12)	0.114 (2)	0.0377 (15)	−0.0038 (13)	0.0058 (11)	−0.0082 (15)
C29	0.0391 (12)	0.1030 (19)	0.0486 (15)	−0.0098 (12)	−0.0013 (11)	0.0014 (14)
C30	0.0467 (13)	0.0750 (16)	0.0327 (16)	−0.0054 (10)	−0.0042 (12)	0.0004 (10)
C31	0.0447 (13)	0.108 (2)	0.0363 (16)	−0.0031 (12)	−0.0027 (12)	−0.0025 (13)
C32	0.0672 (14)	0.133 (3)	0.0432 (15)	−0.0126 (16)	0.0118 (13)	0.0041 (17)
C33	0.0753 (17)	0.112 (2)	0.0479 (17)	−0.0106 (16)	0.0049 (14)	0.0165 (17)
C34	0.084 (2)	0.125 (3)	0.096 (2)	−0.0012 (19)	−0.0010 (18)	0.034 (2)
C35	0.127 (3)	0.118 (4)	0.147 (4)	0.027 (3)	0.024 (3)	0.053 (3)
C36	0.171 (4)	0.105 (3)	0.097 (3)	−0.002 (3)	0.026 (3)	0.011 (2)
C37	0.146 (4)	0.123 (3)	0.099 (3)	−0.004 (3)	−0.008 (3)	0.008 (3)
C38	0.101 (2)	0.113 (3)	0.084 (2)	−0.0056 (19)	−0.0081 (18)	0.004 (2)
N1	0.0406 (10)	0.0901 (14)	0.0408 (15)	−0.0008 (8)	−0.0098 (10)	0.0026 (10)
N2	0.0325 (9)	0.0872 (14)	0.0405 (14)	0.0011 (7)	−0.0005 (10)	0.0027 (8)
N3	0.0336 (10)	0.0979 (15)	0.0360 (15)	−0.0022 (8)	0.0001 (10)	−0.0017 (9)
N4	0.0423 (11)	0.0982 (15)	0.0347 (14)	−0.0044 (8)	−0.0038 (10)	−0.0027 (9)
O1	0.0577 (12)	0.192 (2)	0.0431 (15)	0.0075 (11)	−0.0041 (12)	−0.0181 (12)
O2	0.0582 (13)	0.223 (3)	0.0443 (16)	0.0125 (12)	−0.0046 (12)	0.0058 (14)
O3	0.0593 (10)	0.1132 (14)	0.0402 (11)	0.0041 (9)	0.0053 (8)	−0.0093 (9)
O4	0.0389 (8)	0.1202 (15)	0.0733 (12)	0.0012 (8)	−0.0007 (8)	−0.0006 (11)
O5	0.0535 (9)	0.1195 (15)	0.0448 (11)	−0.0020 (9)	−0.0080 (9)	−0.0004 (10)
O6	0.0649 (10)	0.1105 (14)	0.0373 (11)	0.0007 (9)	0.0047 (8)	−0.0088 (10)
O7	0.0366 (9)	0.174 (2)	0.0751 (13)	−0.0020 (10)	−0.0013 (9)	−0.0119 (13)
O8	0.0517 (9)	0.1655 (18)	0.0449 (11)	0.0017 (10)	−0.0126 (9)	−0.0159 (11)

### Geometric parameters (Å, º)

**Table:**

C6—C1	1.363 (5)	C16—C20	1.541 (6)
C6—C5	1.397 (5)	C17—C18	1.396 (5)
C19—C1	1.513 (6)	C21—C22	1.351 (6)
C39—C21	1.534 (6)	C21—C26	1.373 (5)
C40—C36	1.524 (6)	C22—C23	1.392 (5)
C1—C2	1.378 (6)	C23—C24	1.367 (4)
C2—C3	1.396 (5)	C24—C25	1.383 (5)
C3—C4	1.396 (4)	C24—C27	1.511 (4)
C4—C5	1.386 (4)	C25—C26	1.403 (6)
C4—C7	1.506 (4)	C27—C28	1.559 (4)
C7—C8	1.553 (3)	C28—C31	1.506 (3)
C8—C11	1.513 (3)	C28—C29	1.518 (4)
C8—C9	1.521 (3)	C28—C32	1.564 (4)
C8—C12	1.559 (4)	C29—O7	1.214 (3)
C9—O5	1.216 (3)	C29—N4	1.362 (4)
C9—N2	1.357 (3)	C30—O6	1.207 (3)
C10—O3	1.218 (3)	C30—N3	1.360 (3)
C10—N1	1.363 (3)	C30—N4	1.371 (3)
C10—N2	1.380 (4)	C31—O8	1.221 (3)
C11—O4	1.214 (2)	C31—N3	1.361 (4)
C11—N1	1.360 (3)	C32—C33	1.494 (4)
C12—C13	1.500 (4)	C33—C34	1.380 (4)
C13—C18	1.378 (4)	C33—C38	1.393 (4)
C13—C14	1.386 (4)	C34—C35	1.403 (6)
C14—C15	1.372 (5)	C35—C36	1.380 (6)
C15—C16	1.374 (5)	C36—C37	1.368 (6)
C16—C17	1.387 (6)	C37—C38	1.358 (6)
			
C1—C6—C5	122.8 (4)	C26—C21—C39	118.6 (5)
C6—C1—C2	116.1 (4)	C21—C22—C23	121.0 (4)
C6—C1—C19	121.7 (5)	C24—C23—C22	121.9 (3)
C2—C1—C19	122.2 (5)	C23—C24—C25	117.3 (3)
C1—C2—C3	122.8 (4)	C23—C24—C27	121.4 (3)
C4—C3—C2	120.4 (4)	C25—C24—C27	121.2 (3)
C5—C4—C3	116.8 (3)	C24—C25—C26	120.4 (4)
C5—C4—C7	122.4 (3)	C21—C26—C25	121.0 (4)
C3—C4—C7	120.8 (3)	C24—C27—C28	114.9 (2)
C4—C5—C6	121.0 (3)	C31—C28—C29	113.1 (2)
C4—C7—C8	115.3 (2)	C31—C28—C27	110.0 (2)
C11—C8—C9	113.1 (2)	C29—C28—C27	108.5 (2)
C11—C8—C7	109.4 (2)	C31—C28—C32	107.7 (2)
C9—C8—C7	109.44 (19)	C29—C28—C32	107.7 (2)
C11—C8—C12	107.91 (19)	C27—C28—C32	109.8 (2)
C9—C8—C12	107.17 (18)	O7—C29—N4	119.8 (2)
C7—C8—C12	109.7 (2)	O7—C29—C28	121.1 (2)
O5—C9—N2	119.9 (2)	N4—C29—C28	119.10 (19)
O5—C9—C8	121.6 (2)	O6—C30—N3	122.4 (2)
N2—C9—C8	118.5 (2)	O6—C30—N4	122.0 (2)
O3—C10—N1	122.3 (3)	N3—C30—N4	115.6 (2)
O3—C10—N2	121.7 (2)	O8—C31—N3	120.2 (2)
N1—C10—N2	116.0 (2)	O8—C31—C28	121.7 (2)
O4—C11—N1	120.5 (2)	N3—C31—C28	118.0 (2)
O4—C11—C8	120.5 (2)	C33—C32—C28	114.8 (2)
N1—C11—C8	119.00 (18)	C34—C33—C38	117.0 (3)
C13—C12—C8	115.6 (2)	C34—C33—C32	122.5 (3)
C18—C13—C14	117.2 (3)	C38—C33—C32	120.5 (3)
C18—C13—C12	121.5 (2)	C33—C34—C35	121.6 (4)
C14—C13—C12	121.3 (3)	C36—C35—C34	120.3 (4)
C15—C14—C13	122.2 (3)	C37—C36—C35	117.0 (4)
C14—C15—C16	121.0 (4)	C37—C36—C40	124.4 (6)
C15—C16—C17	117.8 (4)	C35—C36—C40	118.5 (6)
C15—C16—C20	121.1 (4)	C38—C37—C36	123.6 (4)
C17—C16—C20	121.1 (5)	C37—C38—C33	120.5 (3)
C16—C17—C18	121.0 (4)	C11—N1—C10	126.2 (2)
C13—C18—C17	120.8 (3)	C9—N2—C10	126.29 (19)
C22—C21—C26	118.3 (5)	C30—N3—C31	127.6 (2)
C22—C21—C39	123.1 (5)	C29—N4—C30	125.7 (2)
			
C5—C6—C1—C2	−1.3 (5)	C39—C21—C26—C25	179.0 (4)
C5—C6—C1—C19	−179.7 (3)	C24—C25—C26—C21	0.0 (6)
C6—C1—C2—C3	2.7 (6)	C23—C24—C27—C28	−90.5 (3)
C19—C1—C2—C3	−178.9 (3)	C25—C24—C27—C28	93.0 (3)
C1—C2—C3—C4	−2.0 (5)	C24—C27—C28—C31	60.9 (3)
C2—C3—C4—C5	−0.2 (4)	C24—C27—C28—C29	−63.3 (3)
C2—C3—C4—C7	179.4 (3)	C24—C27—C28—C32	179.3 (2)
C3—C4—C5—C6	1.5 (4)	C31—C28—C29—O7	−171.5 (3)
C7—C4—C5—C6	−178.1 (3)	C27—C28—C29—O7	−49.2 (3)
C1—C6—C5—C4	−0.7 (5)	C32—C28—C29—O7	69.6 (3)
C5—C4—C7—C8	82.6 (3)	C31—C28—C29—N4	9.8 (3)
C3—C4—C7—C8	−97.0 (3)	C27—C28—C29—N4	132.0 (2)
C4—C7—C8—C11	59.5 (3)	C32—C28—C29—N4	−109.2 (3)
C4—C7—C8—C9	−64.9 (3)	C29—C28—C31—O8	172.4 (3)
C4—C7—C8—C12	177.7 (2)	C27—C28—C31—O8	50.9 (3)
C11—C8—C9—O5	−172.6 (2)	C32—C28—C31—O8	−68.7 (3)
C7—C8—C9—O5	−50.3 (3)	C29—C28—C31—N3	−9.4 (3)
C12—C8—C9—O5	68.6 (3)	C27—C28—C31—N3	−130.8 (3)
C11—C8—C9—N2	10.0 (3)	C32—C28—C31—N3	109.5 (3)
C7—C8—C9—N2	132.3 (2)	C31—C28—C32—C33	−57.5 (3)
C12—C8—C9—N2	−108.8 (2)	C29—C28—C32—C33	64.8 (3)
C9—C8—C11—O4	171.2 (2)	C27—C28—C32—C33	−177.3 (2)
C7—C8—C11—O4	48.9 (3)	C28—C32—C33—C34	86.1 (3)
C12—C8—C11—O4	−70.4 (3)	C28—C32—C33—C38	−95.3 (3)
C9—C8—C11—N1	−9.5 (3)	C38—C33—C34—C35	3.6 (5)
C7—C8—C11—N1	−131.8 (2)	C32—C33—C34—C35	−177.7 (3)
C12—C8—C11—N1	108.9 (2)	C33—C34—C35—C36	−2.5 (6)
C11—C8—C12—C13	−66.5 (2)	C34—C35—C36—C37	−0.3 (7)
C9—C8—C12—C13	55.6 (3)	C34—C35—C36—C40	−179.1 (4)
C7—C8—C12—C13	174.32 (19)	C35—C36—C37—C38	1.9 (7)
C8—C12—C13—C18	89.4 (3)	C40—C36—C37—C38	−179.4 (4)
C8—C12—C13—C14	−89.2 (3)	C36—C37—C38—C33	−0.6 (7)
C18—C13—C14—C15	−0.7 (4)	C34—C33—C38—C37	−2.1 (5)
C12—C13—C14—C15	178.0 (3)	C32—C33—C38—C37	179.2 (3)
C13—C14—C15—C16	0.2 (5)	O4—C11—N1—C10	−177.7 (2)
C14—C15—C16—C17	0.5 (6)	C8—C11—N1—C10	3.0 (3)
C14—C15—C16—C20	178.6 (4)	O3—C10—N1—C11	−177.6 (2)
C15—C16—C17—C18	−0.6 (6)	N2—C10—N1—C11	3.6 (3)
C20—C16—C17—C18	−178.7 (4)	O5—C9—N2—C10	178.3 (2)
C14—C13—C18—C17	0.6 (4)	C8—C9—N2—C10	−4.3 (3)
C12—C13—C18—C17	−178.1 (3)	O3—C10—N2—C9	178.3 (2)
C16—C17—C18—C13	0.0 (6)	N1—C10—N2—C9	−2.9 (3)
C26—C21—C22—C23	1.2 (6)	O6—C30—N3—C31	−178.9 (3)
C39—C21—C22—C23	−179.4 (4)	N4—C30—N3—C31	3.3 (3)
C21—C22—C23—C24	0.8 (6)	O8—C31—N3—C30	−178.5 (2)
C22—C23—C24—C25	−2.4 (5)	C28—C31—N3—C30	3.3 (4)
C22—C23—C24—C27	−179.0 (3)	O7—C29—N4—C30	177.3 (2)
C23—C24—C25—C26	1.9 (5)	C28—C29—N4—C30	−3.9 (4)
C27—C24—C25—C26	178.5 (3)	O6—C30—N4—C29	179.2 (2)
C22—C21—C26—C25	−1.6 (6)	N3—C30—N4—C29	−2.9 (3)

### Hydrogen-bond geometry (Å, º)

Cg is the centroid of the C13–C18 benzene ring.

**Table:**

*D*—H···*A*	*D*—H	H···*A*	*D*···*A*	*D*—H···*A*
N1—H*N*1···O1	0.86	1.94	2.787 (4)	167
O1—H1*A*···O3^i^	0.84 (3)	2.13 (3)	2.949 (3)	162
O1—H1*B*···O5^ii^	0.90 (3)	1.90 (3)	2.794 (4)	175
N2—H*N*2···O4^iii^	0.86	1.94	2.767 (2)	162
O2—H2*A*···O8^iv^	0.86 (6)	1.88 (6)	2.739 (4)	177
O2—H2*B*···O6^v^	0.82 (3)	2.16 (3)	2.961 (3)	169
N3—H*N*3···O7^vi^	0.86	1.90	2.739 (2)	164
N4—H*N*4···O2	0.86	1.92	2.761 (4)	166
C22—H22···*Cg*^vii^	0.93	2.97	3.5693	124

Symmetry codes: (i) *x*+1/2, −*y*+2, *z*; (ii) −*x*+2, −*y*+2, *z*+1/2; (iii) *x*−1/2, −*y*+2, *z*; (iv) −*x*+3/2, *y*, *z*−1/2; (v) *x*−1/2, −*y*+1, *z*; (vi) *x*+1/2, −*y*+1, *z*; (vii) −*x*+1, −*y*, *z*+1/2.
